# Rock River Medical Society

**Published:** 1875-11

**Authors:** F. E. Sherman

**Affiliations:** Secretary


### ROCK RIVER MEDICAL SOCIETY.

*Meeting, September* 27, 1875.

(Reported by F. E. Sherman, M.D., Secretary.)

The Rock River Medical Society met pursuant to adjournment, at New Cassell, Fond du Lac county, Wis., and was called to order at 10.30 A.M. President Senn in the chair.

Dr. Marston, of New Cassell, reported an interesting case of ruptured uterus, in which the patient recovered in the first instance, dying from a repetition of the accident in a subsequent labor. The remarkable points of the case were the recovery after the first rupture, and prolapse of a portion of omentum through the rent, which was subsequently removed by the process of suppuration.

Dr. Senn, of Milwaukee, then presented two pathological specimens: excised head of the femur, and tibia and fibula at the ankle joint, relating the cases.

The regular discussion of the subject reported on, regarded immovable fracture dressings, in which all the members took part. Numerous varieties of permanent dressings were mentioned—the general opinion prevailing that in ordinary fractures, as an immediate dressing, they were somewhat objectionable, while in some special cases, and as a later means of support, they might be used to great advantage.

The committee on place and time of next meeting, reported in favor of Hartford, Tuesday, October 19. Subject for discussion: "The duties of a physician in natural labor."

On motion, the Society adjourned according to the report of committee.

---

INFLUENCE OF BROMIDE OF POTASSIUM ON THE MENSTRUATION. *Cordes.*—A delicate but healthy young lady, who menstruated every three weeks, was given half a grain of bromide of potassium daily for one week preceding the expected menstruation. Whereupon the menses appeared every four weeks, but they returned in tri-weekly intervals as soon as the bromide was discontinued.—*Bull. de Thérapie; Memorabilien*, xx. 7.

